# Supplementary material for: Post-Transplant Cyclophosphamide-Based Prophylaxis and Its Impact on Infectious Complications and Immune Reconstitution According to Donor Type
Source: Cancers (Basel). 2025 Mar 26;17(7):1109. doi: 10.3390/cancers17071109 (PMC11987969; doi:10.3390/cancers17071109)
Supplement: Supplementary file 1 [file cancers-17-01109-s001.zip › cancers-3504963-supplementary.pdf]

General criteria for pre-transplant organ function include left ventricular ejection fraction ≥40% without significant pre-existing cardiac disease or uncontrolled arrhythmia; pulmonary function testing demonstrating diffusing capacity of carbon monoxide >40% predicted; normal/stable kidney function; and liver functions tests showing total bilirubin <2.5 times normal with transaminases <3 times the upper limit of normal.

High resolution DNA typing for HLA-A, -B, -C, -DRB1, and -DQB1 was conducted in all recipients and donors. 9/10 MMUD was defined as a donor-recipient match for 4 out of the following 5 alleles: HLA-A, -B, -C, -DRB1, and -DQB1. Donor selection followed the subsequent algorithm: an HLA-matched sibling donor (MSD) followed by a 10/10 HLA-matched unrelated donor (MUD) are preferred upfront. In the absence of a 10/10 HLA-matched donor, alternative stem cell sources as 9/10 MMUD or haploidentical donors are considered the third and fourth choices at our institution.

Engraftment after allo-HCT was defined as the presence of an absolute neutrophil count greater than $\geq 0.5 \times 10^9/L$ on the first of three consecutive days. Platelet recovery was defined as a sustained platelet count $> 20 \times 10^9/L$ (1st of 3 days) without platelet transfusion for 7 days. Primary graft failure was defined as peripheral blood ANC $< 0.5 \times 10^9/L$ by day+28 after allo-HSCT in the absence of relapse and secondary graft failure was defined as loss of donor chimerism (<5% donor cells), in whole blood, after initial engraftment and recurrent ANC $< 0.5 \times 10^9/L$ (35).
